# Pyorrhœa Alveolaris

**Published:** 1894-04

**Authors:** 


### PYORRHŒA ALVEOLARIS.

It will be evident to the readers of the present number that Professor Peirce's paper on this subject, published in the January issue of this journal, has created a wide interest. It is not surprising that it has elicited a diversity of opinion, for from the character of the paper this was to be expected. We devote a large portion of our space in this number to this subject as viewed by various minds, and while the results may not equal the expenditure of words, the effect must be to force to a more exact examination of this difficult pathological problem.

